# Simian varicella virus infection in rhesus macaques is associated with amyloid deposition in multiple organs and arteries and production of an amyloidogenic state

**DOI:** 10.1002/alz.090842

**Published:** 2025-01-09

**Authors:** Ravi Mahalingam, Andrew N. Bubak, Amalia Bustillos, Brittany Feia, Arpita Das, Eileen de Haro, Lara Doyle‐Meyers, Jayme Looper, Christy S. Niemeyer, Diego Restrepo, Maria A. Nagel, Vicki Traina‐Dorge

**Affiliations:** ^1^ University of Colorado School of Medicine, Aurora, CO USA; ^2^ University of Colorado ‐ Anschutz medical school, Aurora, CO USA; ^3^ University of Colorado School of Medicine, Anschutz Medical Cmapus, Aurora, CO USA; ^4^ Tulane National Primate Research Center, Tulane University, Coviington, LA USA; ^5^ LSU School of Veterinary Medicine, Baton Rouge, LA USA

## Abstract

**Background:**

Varicella zoster virus (VZV) is latent in ganglionic neurons in >90% of the world population and reactivates to produce herpes zoster in older adults. Zoster increases dementia risk, of which Alzheimer’s disease (AD) is the most common. However, a critical barrier in studying the mechanisms by which VZV contributes to dementia is that VZV is an exclusively human virus. Simian varicella virus (SVV) is the non‐human primate homolog of VZV that produces the same clinical spectrum of disease from varicella to latency to zoster. Thus, we used the SVV infection model in vivo to examine how infection may recapitulate AD pathology, specifically the induction of amyloidogenic peptides and amyloid deposition, thereby accelerating disease progression.

**Method:**

Rhesus macaques were infected with SVV such that they developed varicella, established virus latency, then developed zoster following immune suppression; serum, tissues, and arteries were harvested across timepoints. Blood was examined for the presence of SVV DNA by PCR from pre‐inoculation to zoster. Serum was analyzed by ELISAs and Thio‐T assays (following an amylin spike) for the presence of amyloidogenic peptides and an amyloid‐promoting environment, respectively. Various tissues and arteries in the periphery and central nervous system were examined by immunohistochemistry for SVV antigen and amyloid.

**Results:**

At the height of SVV viremia, increased amylin was seen and an amyloidogenic environment was induced in serum compared to pre‐inoculation and viremia resolution. SVV antigen co‐localized with amyloid in the periphery (pancreas, GI tract, temporal artery, olfactory epithelium and bulb) and amyloid was found in central nervous system (cerebral arteries).

**Conclusion:**

Varicella infection may accelerate AD progression, in part through increasing the amyloid burden. In particular, the presence of amyloid in cerebral arteries in infected monkeys further support the role of varicella infection in the cerebrovascular disease (including cerebral amyloid angiopathy) seen in AD brains.

